# Topology-Dependent Polymer Stretching and Scission in Solution at Extreme Shear Rates

**DOI:** 10.1021/acspolymersau.5c00105

**Published:** 2025-11-18

**Authors:** Bas G. P. van Ravensteijn, Patrick T. Corona, Anukta Datta, Kexin Dai, Raghida Bou Zerdan, Katie M. Weigandt, Ryan P. Murphy, Craig J. Hawker, Matthew E. Helgeson

**Affiliations:** † Department of Chemical Engineering, 8125 University of California Santa Barbara, Santa Barbara, California 93106, United States; ‡ Mitsubishi Chemical Center for Advanced Materials, 8786 University of California Santa Barbara, Santa Barbara, California 93106, United States; § Center for Neutron Research, 10833 National Institute of Standards and Technology,, Gaithersburg, Maryland 20899, United States; ∥ Materials Department, 2167 University of California, Santa Barbara, Santa Barbara, California 93106, United States

**Keywords:** polymer topology, solution rheology, mechanochemistry, polymer scission, neutron scattering

## Abstract

There has been significant interest in the engineering of polymer topology to control rheology and mechanical stability in dilute solutions for applications involving extreme shear rate flows. However, methods to experimentally probe properties at relevant shear rates (≈ 10^4^–10^6^ s^–1^) have remained *ex situ*, obscuring access to measures of polymer deformation and rheology that would otherwise provide mechanistic insight into the topology-dependent properties that control their behavior in extreme shear flows. In this study, we used novel *in situ* small angle neutron scattering measurements in a capillary rheometer (capillary rheo-SANS) to simultaneously measure solution viscosities and polymer deformations in high shear on a series of chemically homologous topology-defined polymers including linear, randomly branched, and star-shaped molecules. We demonstrate that differences in the onset of chain stretching and shear thinning of these polymers in dilute solution are controlled primarily by differences in their molecular relaxation time. These differences correlate with differences in chain scission inferred from *ex situ* measurements at more extreme shear rates. Together, the results demonstrate a direct coupling between chain deformation and scission, and suggest that the dominant effect of branching as a means to impart resilience against mechanical degradation is through differences in relaxation dynamics due to branching. We anticipate that these results will provide key insights to engineer topology-controlled polymers for rheological modification, mechanical stability, and controlled mechano-chemistry.

## Graphical abstract

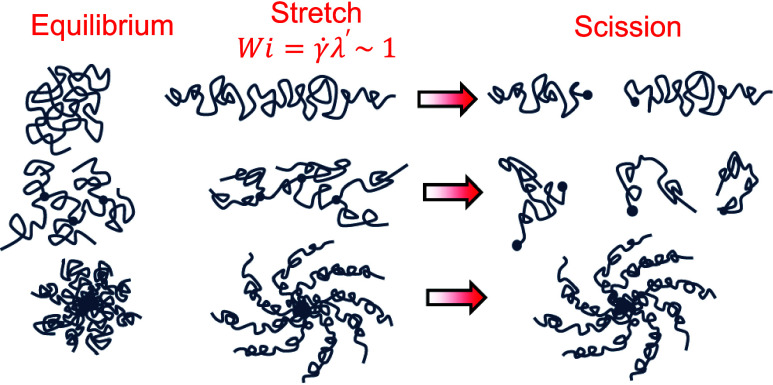

## Introduction

Macromolecules in dilute solution are crucial for controlling the flow and rheology of fluids for a wide array of technologies including lubrication, viscosity regulation, turbulent drag reduction, and flow stability (e.g., in jetting-based printing methods).^1–7^ In these applications, polymers are often engineered to achieve several orthogonal functions including thermorheological control (e.g., temperature-thinning),^8,9^ non-Newtonian response (e.g., shear-thinning or extension-thickening), and mechanical stability, i.e., resistance to mechanically induced molecular scission.^10,11^ Conventionally, polymer chemistry, molecular weight and concentration are used in concert to simultaneously optimize these functions. A key challenge toward this aim is the limited shear rates range of conventional rheometric devices (up to ≈10^3^ s^–1^), whereas the applications noted previously span shear rates in the range of 10^4^–10^6^ s^–1^, posing a significant bottleneck toward the advanced design of rheological modifiers.

More recently, polymer topology has emerged as an orthogonal means to control dilute polymer rheology. In particular, topology has been widely explored as a means of optimizing polymers’ resistance to mechanical degradation.^12–14^ Previous studies suggested that highly branched polymers, such as comb, star-shaped or dendrimeric architectures, exhibit suppressed mechanical scission compared to linear polymers based on *ex situ* measurements of molecular weight after being subjected to uncontrolled extreme shear rate flows (e.g., ultrasonication or homogenization).^15–18^ A key question when comparing properties and performance of topology-defined polymers is the conditions of macromolecular design and deformation under which the effects of topology can be isolated. Some studies compare linear and branched polymers of similar total MW,^19^ whereas others compare molecules with a similar property of interest, e.g., the intrinsic viscosity.^20,21^ In the present work, we will ultimately argue that neither of these previously controlled properties is the relevant one for understanding the influence of topology on molecular deformation and scission.

As a result, it is not yet understood why highly branched topologies resist scission compared to their linear counterparts. In the case of linear polymers, it has long been proposed that mechanical scission occurs due to chain tension accumulated during flow, resulting in enhanced scission in the center of the chain where tension is most concentrated.^15^ For branched polymers, a common hypothesis is thus that macromolecular branching divides molecular tension between covalently adjoined chains, thereby more efficiently distributing elastic stress over the entire macromolecule.^22^ However, confirming this hypothesis would require experiments that directly assess the state(s) of molecular deformation *in situ* during flow and relate that to occurrences of scission, and at relevant strain rates for applications.

In principle, *in situ* small angle neutron scattering (SANS) in flow (“rheo-SANS” measurements) provides an effective means to characterize the state of deformation of polymers in flow.^23,24^ However, the vast majority of devices used in these measurements are either rheometric devices (which, as mentioned above, are limited to shear rates that are irrelevant for most applications where scission occurs), or involve nonhomogeneous straining that prevents precise quantification of molecular deformation in the flow.^25^ Recently, the ability to perform rheo-SANS measurements in relatively idealized high shear flows has been enabled by the development of a capillary rheometer compatible with SANS (capillary rheo-SANS abbreviated as CR-SANS).^26^ Capillary rheo-SANS measurements provide simultaneous measurements of SANS and fluid viscosity at wall shear rates in excess of 10^6^ s^–1^. Along with recent advances in scattering models to quantitatively infer anisotropic macromolecular conformations in flow,^27–30^ CR-SANS is ideal to mechanistically study the influence of macromolecular topology on polymer deformation, rheology, and scission in extreme shear rate flows.

In this work, we adapt these advanced measurements on a series of chemically identical topology-defined polymers in order to assess the dominant effects of topology on molecular deformation and shear thinning rheology at high shear rates (γ̇ ≥ 10^5^ s^–1^). We use these measurements to elucidate the primary mechanisms by which branching imparts enhanced mechanical stability to complex topology-controlled polymers. Ultimately, we show the molecular conformational relaxation time (λ) as a key parameter that dominates both molecular deformation and scission. Although this result is entirely consistent with molecular theories of dilute polymer rheology which predict that steady-state molecular stretching in shear flow is governed by the Weissenberg number, *Wi* = λγ̇; this framework has not yet been applied to interpret experiments on the shear-induced degradation of topology-defined polymers. We specifically hypothesize that polymers of similar molecular weight or intrinsic viscosity but distinct topologies exhibit markedly different relaxation dynamics, leading to different scission outcomes. These differences arise not from variations in molecular tension at a given level of overall stretch, but because highly branched molecules relax more rapidly, thereby reducing their molecular extension under an imposed shear rate. In this sense, the most important way topology influences deformation is through its effect on the longest relaxation time. We test this hypothesis using a systematically designed series of topology-controlled polymers, combining *in situ* capillary rheo-SANS at shear rates below the scission threshold with *ex situ* measurements at elevated shear rates where scission is observed.

## Materials and Methods

### Materials

The topology-defined polymers studied throughout this work are based on statistical copolymers of methyl methacrylate (MMA) and stearyl methacrylate (SMA) (Figure 1), a chemistry that is commonly used for oil-soluble rheology modifiers. The equilibrium solution behavior of these polymers and their performance for thermo-rheological and lubrication control was reported previously.^3,31^ Topological control over the macromolecular structure was achieved using controlled radical polymerization to synthesize linear polymers (*LP*_170_ and *LP*_220_, Table 1, entry 1 and 2) and 8-arm star polymers (*SP*_180_, Table 1, entry 4) according to previous schemes using conditions that minimize coupling between chain ends, ensuring topological control while minimizing dispersity.^3,31^ An additional long-chain randomly branched polymer with a statistical distribution of branch points was obtained by relying on synthetically uncontrolled free-radical polymerization (*BP*_130_, Table 1, entry 3), making this polymer representative of conventional industrial rheological modifiers as a benchmark for the study. The experiments were carried out using 3 wt % polymer solution in toluene (or deuterated-toluene for the neutron scattering measurements). This concentration correspond to *c*/c* values in the range of 1.1–1.6 for the polymers studied, where c* is the overlap concentration estimated from intrinsic viscosity measurements.

**Figure 1:**
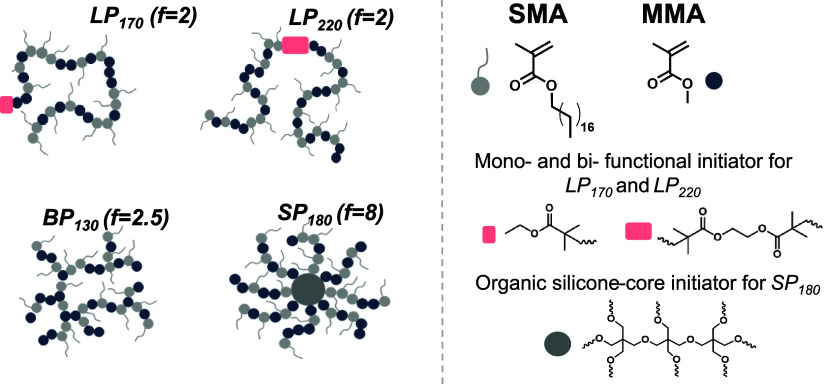
Overview of the poly­(stearyl methacrylate-*co*-methyl methacrylate) (p­(SMA-MMA)) topological library consisting of linear (*LP*_170_ and *LP*_220_), randomly branched (*BP*_130_), and star-shaped (*SP*_180_) polymers. The chemical structures of SMA, MMA and the initiators for linear (red rectangles) and star-shaped (dark gray circle) polymers are depicted on the right side of the figure. The subscripts in the abbreviations of the macromolecules refer to their absolute molecular weight and *f* represents the functionality of the polymer, i.e., the number of branch points.

**Table 1:** Physical Characteristics of the Topology-Defined Poly­(SMA-*co*-MMA) Polymers

**additives**	**abs. MW** [kg/mol]^a^	**PĐI** ^b^	***R***_***g**,**eq***–**GPC**_ [**nm**]^a^	***R***_***g**,**eq***–**SANS**_ [**nm**]^c^	[**η**]_**toluene**_ [**dL**/**g**]^d^	**λ** [μ**s**]^e^	λ* = **2**λ/***f*** [μ**s**]
*LP*_170_	170	1.5	11.6 ± 0.7	11.5 ± 4.9	0.555	2.9 ± 0.06	2.9
*LP*_220_	220	1.4	17.7 ± 1.6	20.1 ± 4.9	0.607	3.6 ± 0.04	3.6
*BP*_130_	130	1.9	10.3 ± 1.6	11.4 ± 4.5	0.449	3.4 ± 0.05	2.72
*SP*_180_	180	1.2	8.0 ± 0.6	8.5 ± 1.1	0.402	2.5 ± 0.08	0.63

a Measured using SEC-MALS, See Supporting Information S1.4.

b Measured using SEC-MALS, See Supporting Information S1.0.

c Measured using capillary rheo-SANS at equilibrium, see Supporting Information S1.7.

d Measured using concentration dependent viscosity data, see Supporting Information S1.2.

e Measured using a Carreau–Yasuda fit to flow-curves, see Supporting Information S1.3.

In the analysis to follow, we identify the number of chains emanating from a branch point as *f*. Thus, for a linear polymer (with two “arms”), *f* = 2 and 8 for the 8-“arm” star polymer. For the randomly branched polymer, *f* is ill-defined. To compare with the other well-defined topologies, we developed a statistical model (refer to Section S3 of the Supporting Information) for the most probable degree of branching of a long-chain randomly branched polymer, which can be used to assign an average functionality of an equivalent star polymer with identical span molecular weight. For the *BP*_130_ polymer studied here, the model produces an estimate of *f* ≈ 2.5. This low value of *f* suggests that the degree of branching in this polymer is quite low. Different target degrees of polymerization were chosen for the polymers of different topologies (*LP*_170_, *BP*_130_, *SP*_180_) to approximately match their intrinsic viscosity (or equivalently their molecular volume). Furthermore, to assess the effect of molecular weight with similar architecture, we chose a longer linear polymer (*LP*_220_) for comparison to the shorter linear polymer (*LP*_170_). The synthesized polymers were characterized to determine their equilibrium properties (Table 1).

### High Pressure Homogenization Setup

*ex situ* shearing experiments were performed using an Avestin Emulsiflex C5 high-pressure homogenizer operating in dynamic valve mode. The operating principle is as follows: liquid samples are pressurized to ∼1500 bar and forced through a valve seat with a narrow aperture (orifice diameter 1.6 mm), after which they expand rapidly to atmospheric pressure at the outlet. The resulting steep pressure gradient produces extremely high velocity gradients within the valve gap. As the fluid accelerates through this constriction, it experiences a combination of intense shear and strong extensional deformations, both of which contribute to the high stresses imposed on the sample in the homogenization zone.

Polymer solutions of 3 wt % in chloroform were injected into the homogenizer by using a luer-lock syringe. After the complete sample volume passed through the device, the sample was reintroduced to start a new homogenization cycle. Samples were withdrawn after 10, 20, and 30 cycles. These samples were analyzed using size exclusion chromatography (SEC) to determine the effect of the homogenization on the molecular weight distribution of the polymers (refer to Supporting Information, S1.5). Before injection into the SEC instrument, the samples were diluted to a polymer concentration of approximately 10 mg/mL with a mixture of chloroform and TEA (0.25%) (mobile phase).

### Capillary RheoSANS (CR-SANS) Setup

*In situ* CR-SANS experiments were performed utilizing a custom-built sample environment described in Murphy et al.^26^ Briefly, a polyimide coated silica capillary (Polymicro) of 100 μm inner diameter and 50 cm length was coiled and mounted inside a custom 3D-printed coil holder sample environment, with pressure sensors attached to both ends to measure the pressure drop across the capillary. Viscosity was then determined using the Hagen–Poiseuille equation. The capillary was driven by high-pressure syringe pumps (Cetoni Nemesys) equipped with 3 mL stainless steel syringes, capable of sustaining a maximum pressure drop of 500 bar. The capillary is coiled to maximize the scattering volume, thereby maximizing the scattering contribution from the deformed polymer chains. The quartz and polyimide tubing material ensures minimum background scattering due to the sample environment. Measurements were delayed for at least 10 min to ensure the flow had reached steady state. SANS scattering measurements were performed using the VSANS instrument at the National Institute of Standards Center for Neutron Scattering (Gaithersburg, MD). Scattering from the sample was collected in the *q*-range from 0.005–0.2 Å^–1^ with the wavelength λ = 5 Å and wavelength spread Δλ/λ = 0.12. The scattering vector *q* is defined as $q=\frac{4\pi }{\lambda }\sin\left(\frac{\theta }{2}\right)$ where θ is the angle at which the neutron is scattered and λ is the neutron wavelength. Measurement times were roughly 4 h at each flow rate to resolve anisotropy. The sample environment was placed in the beam path such that the radiation passes through the coil twice along the diameter. The 2D scattering intensities were corrected for empty cell (only the capillary with the deuterated toluene in it) and incoherent background scattering. Scattering spectra were reduced using standard NCNR protocols with Igor PRO software.

## Results and Discussion

### Probing Scission with *Ex Situ* Measurements

To assess the influence of the series of topology-defined polymers on flow-induced scission, high-pressure homogenization followed by *ex situ* size exclusion chromatography with multiangle light scattering (SEC-MALS) was used to track the molecular weight distribution of the polymer solutions after exposure to extreme shear rates (see Supporting Information Figure S1.3 for SEC chromatograms of the MW distribution after homogenizer cycles). At an operating homogenization pressure of 1500 bar, the flow rate through the high-shear zone of the homogenizer is approximately 40 mL/min. Because the homogenizer employs a dynamic valve orifice, it is difficult to define the exact volume of this shear zone and thereby hampers reliable estimation of the shear rates to which the polymers are exposed. However, using reasonable estimates for the active zone (diameter of 20 μm and length of 1 mm) for a pressure drop of ≈ 1500 bar, the shear rate was estimated to be in the range of (≈ 10^6^–10^7^ s^–1^). Using these same estimates, the residence time in the high-shear region is approximately *t*_res_ ∼ 150 μs. The corresponding Deborah number, *De* = *t*_res_/λ, which compares the straining time scale to the material relaxation time, is estimated to be ∼ 100 in the active zone of the homogenizer. The contraction–expansion geometry of the homogenizer involves hyperbolic straining, i.e., predominantly extensional motion. Thus, in this case, *De* ≫ 1 corresponds to the continuous accumulation of molecular strain at a rate that depends on the Weissenberg number, *Wi*. In other words, in the homogenizer we expect the response of the material to represent the large-strain limit of the polymer deformation, i.e., where molecules reach their finite extensibility en route to scission.

To quantify polymer scission, we tracked the number-average molecular weight (*M*_*n*_) from the SEC chromatograms across several homogenization cycles relative to the unsheared polymer (*M*_*n*,0_) to normalize for differences in polydispersity and shape of the molecular weight distribution as shown in Figure 2. *M*_*n*_ is used because it is a robust indicator of the dominant chain size within the complete size distribution and its shift under scission, without being overly influenced by distribution tails, noise or large uncertainty in its experimental determination. Unfortunately, the number of cycles cannot be converted to a Deborah number representation, as we do not have a reasonable estimate of the strain rate or the residence time in the active zone. The SEC chromatograms for each polymer is shown in Figure S1.3 in the Supporting Information. In agreement with previous *ex situ* studies on mechanical scission of topology-controlled polymers^15,32,33^ , chain scission was most severe for the highest MW linear polymer (*LP*_220_) since it undergoes the most change in *M_n_*. For the star-shaped polymer (*SP*_180_), no apparent decrease in the molecular weight was observed. The randomly branched (*BP*_130_) and shorter linear polymer (*LP*_170_) exhibit behavior in between these two extremes, with *BP*_130_ showing more breakage than *LP*_170_.

**Figure 2:**
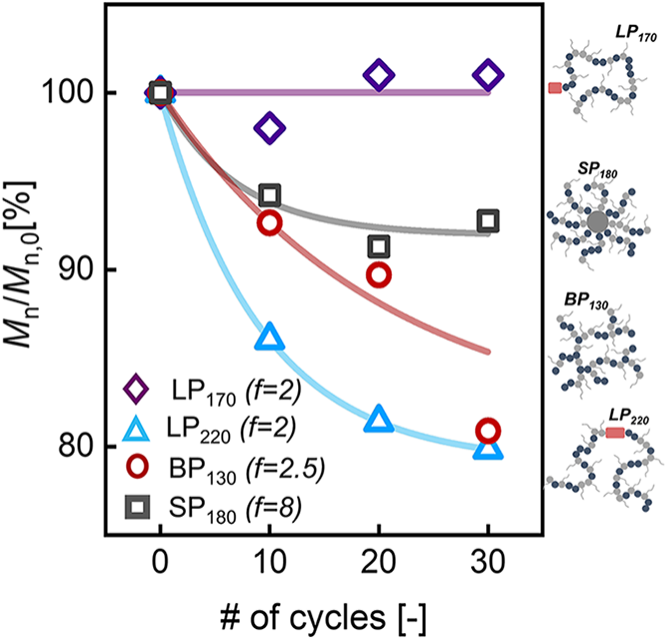
Ratio of the number-averaged molecular weight (*M*_*n*_) to its initial unsheared value (*M*_*n*,0_) of p­(SMA-*co*-MMA) based lubricant additives as a function of shearing time expressed as number of homogenization cycles. *LP*_170_: purple diamond symbols, *LP*_220_: blue triangle symbols, *BP*_130_: maroon circle symbols and *SP*_180_: gray square symbols. Shaded regions are drawn to guide the eye. The data were fitted to an exponential dependence to better represent trends with increasing cycles.

### Polymer Relaxation Dynamics Dictate Polymer Stretch

It is tempting to conclude that the results in Figure 2 confirm previous hypotheses that branching suppresses polymer scission under similar degrees of straining on individual polymer molecules. However, this interpretation assumes that a fixed shear rate produces similar amounts of molecular stretch for the different polymers.^32^ Because shear flow exerts both straining and rotational motion on the polymers, and because different topologies have different conformational dynamics, this need not be the case. Typically, the steady state strain exerted on a polymer fluid in a shear-dominant flow (like in the capillary rheometer device) is nominally controlled by the Weissenberg number, *Wi* = λγ̇, which sets the steady-state molecular strain on the material. By contrast in an extension dominated flow (like in the homogenizer), the accumulated strain is set by a combination of Weissenberg number and Deborah number.

To this end, the relaxation times of the polymers at 3 wt % in toluene were estimated from a Carreau–Yasuda fit to the steady-shear rheology obtained from the capillary rheometer (see Table 1, column 7 for the experimental values of λ). The details of the fitting algorithm and comparison of the fitted relaxation time to that obtained using a Zimm model for a high molecular weight linear polymer in a good solvent^34,35^ are outlined in Section S1.3 of the Supporting Information. A small deviation between the measured and theoretically estimated values of λ was observed for the randomly branched polymer. We expect this, since the uncontrolled and ill-defined branching structure of *BP*_130_ would be expected to exhibit the largest deviations from the Zimm theory.

Due to clear differences in their relaxation behavior, we therefore expect the different topology-defined polymers to exhibit different degrees of molecular stretching, and more detailed characterization of their shear-induced deformation is needed to understand the observed topology-dependent differences in shear-induced scission. Recently, molecular dynamics simulations were used to explore the effect of chain architecture on the structure, dynamics, and rheology of model star, linear and ring polymers in shear flow.^12^ It was found that universal behavior was followed when the longest relaxation time was scaled by the number of arms, *f*. Regardless of the topology, the onset of nonlinear shear-induced effects occurred at a topologically corrected Weissenberg number, ${\mathrm{Wi}}^{*}=\frac{\mathrm{Wi}}{f}=\mathrm{\gamma ̇}\lambda^{*}=\mathrm{\gamma ̇}\frac{2\lambda }{f}∼1$ , where λ* is the topologically corrected relaxation time. Here, the factor of 2 is used to normalize λ* to that of an equivalent linear polymer with the same span molecular weight as a branched polymer. Brownian dynamics simulations of star polymers revealed a similar collapse of viscosity under shear and extensional flow for stars with similar arm lengths and varying total molecular weights.^36^ This implies that in the limit of long noninteracting polymer segments, the longest relaxation time will be equal to that of the longest continuous linear strand (the so-called “span molecular weight”). Thus, to isolate the effects of topology one must compare polymers with approximately the same longest relaxation time. Following this reasoning, we scaled the longest relaxation times obtained from Carreau–Yasuda fits to the steady-shear rheology by the functionality *f* to produce a topologically corrected relaxation time, $\lambda^{*}=\frac{2\lambda }{f}$ (Table 1, column 8).

### Probing Polymer Deformation In Situ using Capillary-RheoSANS

To probe polymer conformational changes as they undergo deformation at moderate strain rates and therefore test the validity of using the topology-corrected relaxation time to predict the onset of strong polymer stretch in flow, *in situ* capillary rheo-SANS experiments were performed. The residence time, *t*_res_, at the highest flow rate probed (6 mL/min) for the 100 μm capillary of 50 cm length was estimated to be approximately 40 ms. Since these residence times greatly exceed the polymer relaxation times (which are of the order of 4 μs), we assume that for all flow rates probed, we observe the material response under steady-state straining. It is important to note that the steady-state response differs between the CR-SANS device and the high-pressure homogenizer due to both the magnitude and type of deformation imposed. The homogenizer subjects polymers to strain rates at least 2 orders of magnitude higher than those attainable in the capillary rheometer and generates predominantly extensional flow, which strongly stretches chains and promotes scission. In contrast, the capillary rheometer produces mainly shear flow comprising both straining and rotational components, leading to tumbling–stretching cycles rather than sustained chain stretch.^37^ Although it would be ideal, directly probing scission in the capillary rheo-SANS setup is not feasible since the maximum accessible shear rates due to pump pressure limitations are at least an order of magnitude lower than those encountered in the homogenizer. Nonetheless, the CR-SANS measurements remain essential, as they enable probing of nonlinear chain deformation under high but sub-scission shear rates, providing critical insight into how molecular topology influences relaxation dynamics and deformation pathways that ultimately can be linked to the onset of scission observed in the homogenizer.

The polymer solutions (same concentration as the scission measurements) were pumped through the capillary at flow rates, *Q* of 0, 1.5, 3, and 6 mL/min. Assuming fully developed Poiseuille flow, the wall shear rate (γ̇_wall_) can be estimated using, ${\mathrm{\gamma ̇}}_{\mathrm{wall}}=\frac{32Q}{\pi D^{3}}$ yielding γ̇_wall_ of 0, 0.26, 0.51, and 1 × 10^6^ s^–1^ respectively. At these shear rates, the achievable *Wi* (=λγ̇_wall_) at the wall varies in the range of 0–5 throughout the topological library, which is sufficient to probe the onset of significant chain stretching (*Wi* ∼ 1). We emphasize that under these experimental conditions, no irreversible changes to the equilibrium scattering were observed for any of the polymers after cessation of flow (see Supporting Information Figure S1.4 for SEC-chromatograms of samples before and after shearing in the capillary), indicating no evidence of chain scission. The absence of scission can be attributed to the comparatively lower strain rates in the capillary, combined with the shear-rate gradient across its cross-section (maximal at the wall and zero at the center)^19^ as well as the shear-dominated flow field, which is less effective than extensional flows at sustaining large, steady-state polymer stretching. Additionally, Murphy et al. previously used a Mooney analysis involving capillary diameter-dependent measurements to show that wall slip in the device is minimal for weakly entangled polymer solutions (∼3% for 100 μm capillaries).^26^ Given that our dilute polymer solutions have coil sizes (*R*_*g*_ ∼ 10 nm) far smaller than the capillary diameter, we conclude that wall-slip effects can be safely neglected in our analysis.

We analyzed the CR-SANS data using an extended Guinier–Maxwell model, a scattering framework developed for Hookean dumbbell polymers deforming in capillary shear flow. This model extends the original Guinier–Maxwell description, which applies to simple shear, by accounting for the nonuniform shear rate profile across the capillary. The development of both models is outlined in Supporting Information Sections S2.1 and S2.2. The Guinier–Maxwell model is a coarse-grained description based on Hookean dumbbells meant to describe the low-*q* scattering from deformed polymers, and does not explicitly account for molecular topology such as branching. We have recently developed a moments based framework that is able to describe finer details of the polymer segment distribution.^30^ Unfortunately, the present CR-SANS data are not sufficiently well-resolved at larger *q* values to apply this analysis. Nevertheless, the fits obtained from the Guinier-Maxwell model serve as effective measures of large-scale coil deformation. The model reproduces the qualitative trends observed experimentally and thus provides a useful framework for comparison across polymer topologies. The resulting intensity from the extended Guinier–Maxwell framework is given as follows,

1 $$\mathrm{I̅}\left(q_{x},q_{y}\right)=I\left(0\right)\left(1+\frac{4}{{\mathrm{Wi}}_{\mathrm{wall}}^{2}}\int_{0}^{{\mathrm{Wi}}_{\mathrm{wall}}}\mathrm{Wi}\cdot \exp\left(-\frac{\langle R_{g}^{2}\rangle_{\mathrm{eq}}\left(q_{x}^{2}+q_{y}^{2}\left(1+2{\mathrm{Wi}}^{2}\right)\right)}{3}\right)\times I_{0}\left(\frac{2}{3}\mathrm{Wi}\langle R_{g}^{2}\rangle_{\mathrm{eq}}q_{x}q_{y}\right)d\mathrm{Wi}\right)$$

Here, *q*_*x*_ and *q*_*y*_ are the scattering vector components in the velocity and velocity-gradient directions, respectively, *I*(0) is the forward scattering intensity at *q* → 0 (which is assumed constant for a given polymer), *Wi*_wall_ represents the Weissenberg number at the capillary wall calculated using γ̇_wall_ and *I*_0_ is the modified Bessel function of the first kind. The equilibrium radii of gyration in the absence of flow (⟨*R*_*g*_^2^⟩_eq_) were extracted following a Guinier analysis on one-dimensional scattering profiles recorded in the capillary device at no flow (see Supporting Information S1.7). The integral over *Wi* represents a convolution of the scattering over the known linear variation of shear stress encountered by the fluid in Poiseuille flow.^26^ We emphasize that this approach could be applied to other flow sample environments that suffer from inhomogeneous flow fields.^38–40^ eq 1 is applied in the Guinier regime (*qR*_g_ ≤ 1), where the scattering model is assumed to be valid for all *Wi*_wall_ and chain topologies included in this study.

Representative CR-SANS scattering patterns are reported in Figure 3 for *LP*_220_. The raw scattering patterns (Figure 3a) contain anisotropic features regardless of the shear rate due to scattering from the coiled capillary tubing. By subtracting the properly scaled scattered intensity of a solvent-filled capillary (constitutes as the empty cell in background subtraction), the signal corresponding to the polymer could be isolated^26^ (Figure 3b). In the absence of flow, isotropic scattering patterns were obtained (Figure 3c, 0 mL/min), as expected from the equilibrium conformation of the polymer chains. In the Poiseuille flow considered in this work, the radii of gyration of the chains are expected to increase along the velocity direction, leading to a decrease in scattering intensity in the *q*_*y*_ direction. Qualitatively, we find this signature in the corrected scattering patterns in some of the sheared samples (Figure 3c (top), 6 mL/min) where the scattering intensity seems to have flattened in the flow direction. To better assess this anisotropy when convoluted with the high degree of measurement noise, we generate simulated scattering data with the extended Guinier-Maxwell model using eq 1 for each polymer at each flow rate. The resulting scattering patterns emphasize the underlying anisotropy (Figure 3c (bottom)) once the large level of noise in the measured patterns is removed.

**Figure 3:**
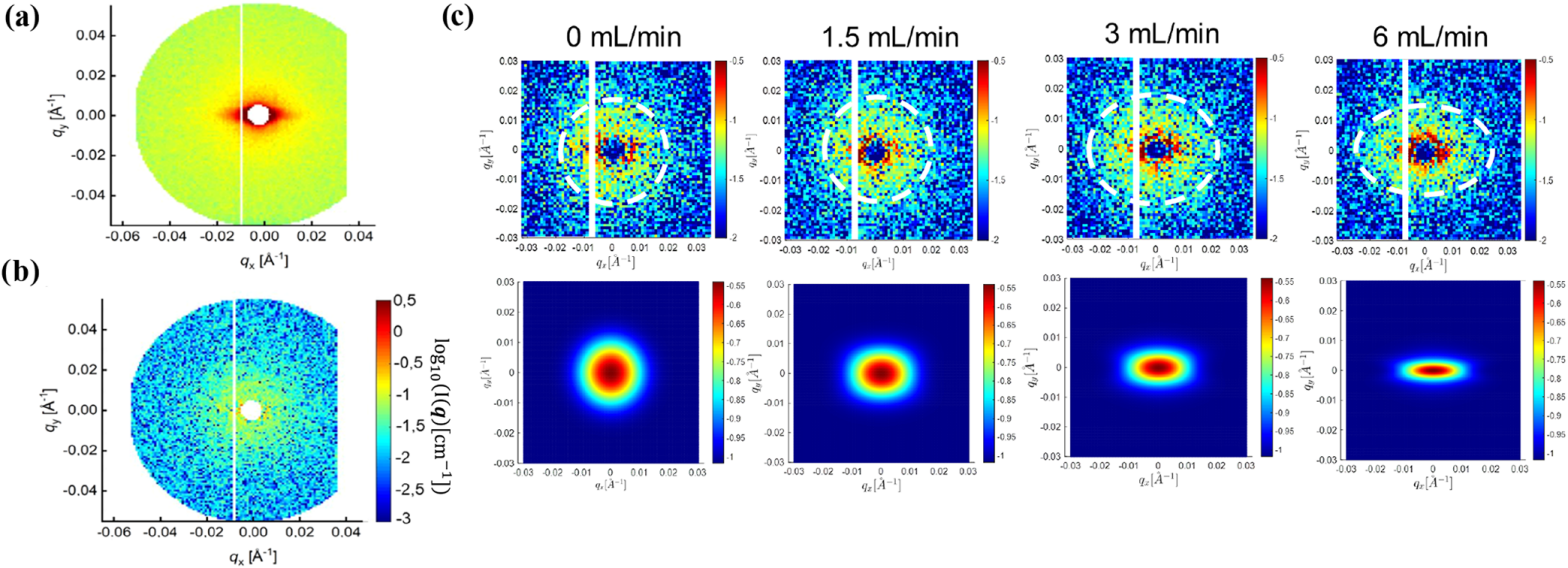
(a) Raw measured scattering pattern of *LP*_220_ polymer at equilibrium in the CR-SANS sample environment. (b) Corrected scattering pattern after subtraction of scattering pattern with solvent in the capillary. (c) (top) Corrected 2D scattering patterns of the *LP*_220_ polymer showing increasing anisotropy (bottom) 2D scattering patterns predicted by the scattering model (eq 1) to emphasize the differences in anisotropy with increasing flow rate.

### Linking Rheological Response to Stretching Behavior

The steady-shear rheology of the polymers was measured using the capillary rheometer and is presented in Figure 4a as the normalized viscosity, η/η_0_ (where η denotes the viscosity at a given flow rate and η_0_ the zero-shear viscosity), plotted against the Weissenberg number, *Wi* = γ̇_wall_λ. The data collapse shows that the onset of non-Newtonian shear thinning for both the linear and star polymers coincides at *Wi* ∼ 1, as expected. This shear thinning is expected when the deformation rate is strong enough to stretch and align polymer chains in the flow direction. In contrast, the branched polymer exhibits a delayed onset, requiring higher shear rates to align in flow before shear thinning can occur. The error bars shown in (Figure 4a) represent the standard deviation of the pressure-drop values measured over the acquisition time at each flow rate.

**Figure 4:**
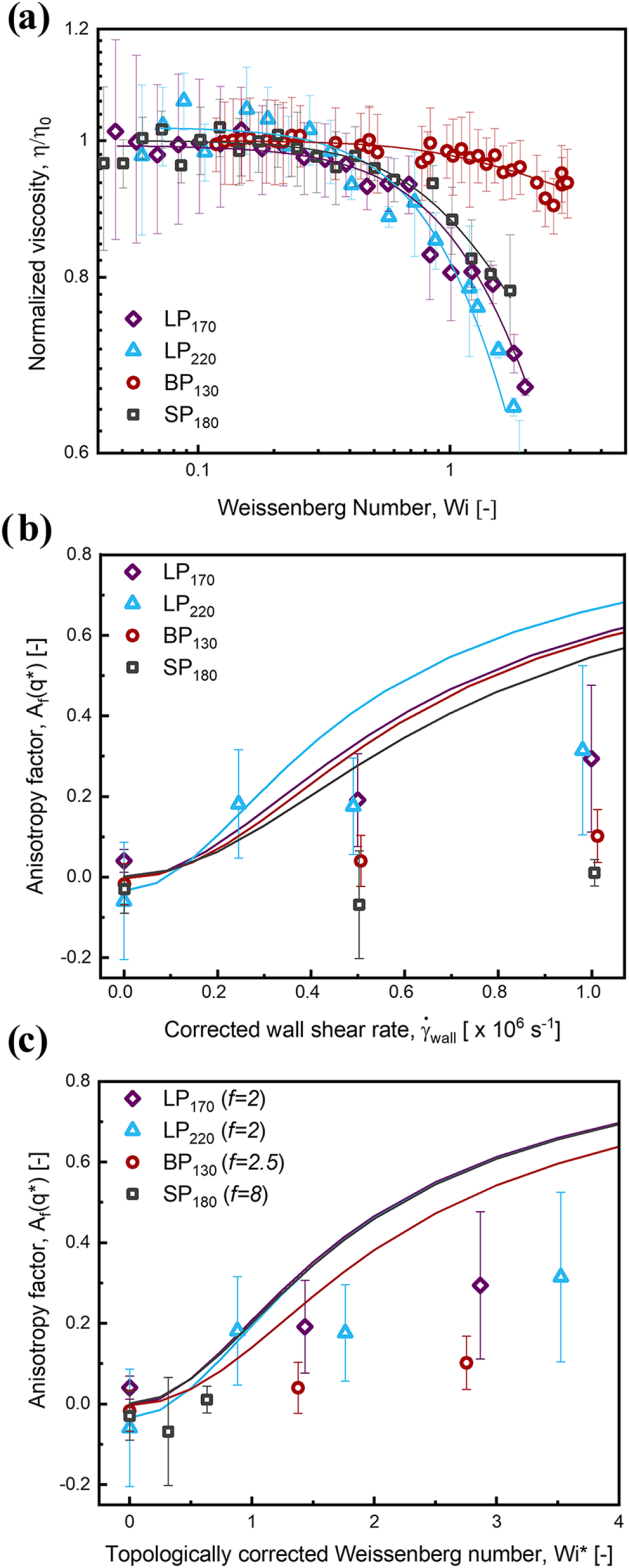
(a) Normalized viscosity (normalized by individual zero-shear viscosity, η_0_) as a function of Wi indicating onset of shear thinning is at approximately *Wi* ∼ 1 (b) Anisotropy factor, *A*_*f*_(q*) as a function of shear rate (γ̇) and (c) *A*_*f*_(q*) as a function of Weissenberg number (*Wi*) for the different polymers. Solid lines are Carreau–Yasuda fits in (a) and *A*_*f*_(q*) calculated at discrete *Wi* using the Guinier-Maxwell model for a Hookean dumbbell polymer with the same *R*_g,eq_ as the polymers studied in this work.

To enable a more direct comparison between the CR-SANS measurements and the Guinier–Maxwell scattering model, we used a scalar parametrization of the annular-averaged scattering intensity, *I*(*q*, θ), known as the anisotropy factor *A*_*f*_(q*). Here, θ denotes the polar angle within the detector plane of an annulus centered at q*. The anisotropy factor, *A*_*f*_(q*) is defined as,

2 $$A_{f}\left(q^{*}\right)=\frac{\int_{0}^{2\pi }\left(I\left(\theta ,q^{*}\right)-\frac{1}{2}I\left(0\right)\right)\cos\left(2\left(\theta -\theta_{0}\right)\right)d\theta }{\int_{0}^{2\pi }\left(I\left(\theta ,q^{*}\right)-\frac{1}{2}I\left(0\right)\right)d\theta }$$

This choice in definition of *A*_*f*_(q*) differs from conventional definitions of an anisotropy factor^41^ through the subtraction of a factor of $\frac{1}{2}I\left(0\right)$ from the forward scattering intensity. This factor removes the *q*-independent incoherent scattering contribution from individual polymer segments,^30^ and thereby ensures that the anisotropy factor increases monotonically with increasing chain stretch. The anisotropy factor, *A*_*f*_, was calculated from the angular dependence of the 2D SANS intensity using 12 bins per 2π radians for the angular discretization. This bin size was chosen as a compromise between statistical precision and angular resolution – fewer bins would obscure the curvature associated with the expected π/2-rad symmetry of the 2D SANS pattern, while a larger number of bins introduced excessive noise and uncertainty in the calculated *A*_*f*_ values. Error bars on *A*_*f*_ were determined from the standard deviation of *A*_*f*_ values from the propagated intensity uncertainties across the angular bins. We note that the value of *A*_*f*_(q*) is highly sensitive to the estimated value of *I*(0). Here, *I*(0) was determined based on the equilibrium fit (data collected in the capillary at 0 mL/min) for each polymer architecture (see Supporting Information, Table S1.2).

Plotting *A*_*f*_(q*) versus shear rate for the linear polymers *LP*_170_ and *LP*_220_ (Figure 4b, blue triangles and purple diamonds, respectively) clearly demonstrates an increase in *A*_*f*_(q*) with increasing wall shear rate over the range of conditions tested. Conversely, the conformation of the star-shaped polymer *SP*_180_ and the randomly branched polymer, *BP*_130_ remains largely unaffected (Figure 4b, gray squares and red circles respectively), with *A*_*f*_(q*) ∼ 0 for the shear rates tested. These measured trends are in qualitative agreement with the *A*_*f*_(q*) calculated from the predicted scattering using eq 1 (Figure 4b, solid lines).

The trends in *A*_*f*_(q*), which serve as a rough indicator of polymer stretch, are in qualitative agreement with the shear stability results from the *ex situ* homogenization experiments (Figure 2). Among the samples, *LP*_220_ exhibits the highest *A*_*f*_(*q*), suggesting the greatest degree of stretching in flow and, consequently, the strongest anisotropy. Consistently, the *ex situ* measurements show that *LP*_220_ also undergoes the most chain scission, reinforcing the interpretation that its enhanced stretch in flow correlates with reduced stability. Conversely, *SP*_180_ displays the lowest *A*_*f*_(q*) at the maximum shear rate probed, consistent with its minimal susceptibility to chain scission. The modest degree of chain alignment for *BP*_130_ compared to the significant chain scission can be explained by the relatively high polydispersity of this polymer.

Through the employed micromechanical scattering model eq 1, the increase in *A*_*f*_(q*) can be directly interpreted as arising from an increase of the nonequilibrium *R*_*g*_ of the polymer due to molecular stretching. The degree of stretching, which can be estimated from eq 1 as *R*_*g*_/*R_g,eq_*, differs according to position in the capillary due to variations in γ̇ as discussed before. By applying eq 1, we estimate that the increase in *A*_*f*_(q*) up to approximately 0.3 observed for the *LP*_170_ and *LP*_220_ polymers corresponds to an average value of *R*_*g*_/*R*_*g*,*eq*_ ∼ 2 when averaged over the cross-section of the capillary. However, when considering only the polymer in the highest shear rate region near the wall, this subset of the polymers represents *R*_*g*_/*R*_*g*,*eq*_ ∼ 5. The observed correlation between shear thinning in viscosity measurements and anisotropy in CR-SANS measurements corroborates and further strengthens the interpretation of the measured scattering anisotropy as arising from polymer stretching and alignment in the flow.

Importantly, plotting *A*_*f*_(*q*) versus ${\mathrm{Wi}}^{*}=\mathrm{\gamma ̇}\frac{2\lambda }{f}$ nearly collapses the experimental data for all of the polymers measured, despite their differences in topology and molecular weight (Figure 4c). This collapse – in which the anisotropy of polymers with shorter relaxation times at higher shear rates fall substantially more within the measurement uncertainty of polymers with longer relaxation times at lower shear rates when plotted versus Wi* relative to either *Wi* or the absolute wall shear rate, γ̇_*w*_^*c*^ – reveals that the key rheological distinctiom between polymers of different topologies is their longest relaxation time. In a steady shear flow, such as in the CR-SANS measurements, the degree of polymer stretching is governed not by the absolute shear rate, but by the Weissenberg number, which measures the ratio of the imposed shear rate to the molecular relaxation time that restores the chain to equilibrium. Consequently, one would not expect molecular-scale properties of polymers with different relaxation times to collapse when plotted against wall shear rate, but such a collapse is expected when they are normalized by the relevant relaxation time through an appropriately defined Weissenberg number. The collapse observed in Figure 4c provides evidence that λ* is indeed the relevant time scale controlling polymer deformation in shear flow. As anticipated, the onset of chain alignment is located at *Wi* ∼ 1, corresponding to the onset of strong shear thinning in the viscosity, and only the linear polymers have sufficiently long relaxation times to exceed this threshold under the probed shear rates. As discussed previously, the ill-defined topology of the branched polymer *BP*_130_ prevents reliable calculation of λ* and hence Wi*; nevertheless, assuming *f* = 2.5 for this polymer produces results consistent with the trends observed for the other, topologically well-defined polymers.

Given this result, our central hypothesis is that the topology-corrected value of the relaxation time, λ* = λ/*f* – which corresponds to the apparent relaxation time associated with the span molecular weight of the polymer – explains the observed differences in both polymer deformation (observed using CR-SANS), as well as scission resulting from that deformation (observed using ex situ molecular weight measurements upon high-pressure homogenization). In other words, topology influences deformation primarily through its effect on the longest relaxation time. Based on this hypothesis, we recommend that future meaningful comparisons across architectures be made for polymers with matched λ*, i.e., matched span molecular weight. Achieving this for the case of star polymers will require the preparation of high MW, low dispersity polymers with *M*_*n*_ that is a factor of *f* larger than an equivalent linear polymer. Doing so remains a considerable synthetic challenge,^42^ and although beyond the present scope this is the subject of ongoing work.

## Conclusions

In summary, we find that polymer topology dictates rheological response and scission behavior primarily through its effect on the longest molecular relaxation time. *Ex situ* homogenizer measurements confirmed the enhanced resistance of branched topologies to mechanical scission in extreme shear flows, while complementary *in situ* capillary rheo-SANS measurements provided direct insight into the state of molecular deformation under shear. Together, these measurements demonstrate that the observed topology-dependent differences in scission arise not from differences in molecular tension distributions, but from the reduced ability of highly branched polymers to sustain deformation due to their shorter relaxation times. This interpretation is reinforced by the reasonable quantitative collapse of topology-dependent scattering data when plotted against a topology-corrected Weissenberg number, which reflects the relaxation dynamics associated with the span molecular weight of the polymer. The implication is that higher shear rates are required to stretch and ultimately break branched macromolecules, establishing span molecular weight as a key molecular design parameter for topology-defined rheology modifiers. Thus, to isolate the effect of chain architecture one should ideally compare different polymer topologies with the same relaxation time, which we find is most closely achieved by matching the span molecular weight, i.e., the longest end-to-end molecular weight of the chain. These findings highlight the value of combining *ex situ* scission measurements with *in situ* structural and rheological characterization to uncover the molecular principles by which topology controls relaxation, deformation, and mechanical stability in high-shear flows. As such, the results highlight the importance of combining careful macromolecular design with direct *in situ* characterization of molecular deformations at high shear rates to aid the rational design of polymer architectures for their application as rheological modifiers.

## Supplementary Material



## Notes

Section S1 provides details regarding polymer synthesis and characterization (intrinsic viscosity, SEC, static SANS and associated modeling). Section S2 provides details of anisotropic scattering models used to interpret capillary rheo-SANS data. Section S3 provides the model used to estimate the functionality parameter *f* for the randomly branched polymer (PDF)

## #

Division of Pharmaceutics, Utrecht Institute for Pharmaceutical Sciences, Faculty of Science, Utrecht University, Utrecht 3584, CG, the Netherlands
