# Supplementary material for: Topology-Dependent Polymer Stretching and Scission in Solution at Extreme Shear Rates
Source: ACS Polym Au. 2025 Nov 18;6(1):194–202. doi: 10.1021/acspolymersau.5c00105 (PMC12903432; doi:10.1021/acspolymersau.5c00105)
Supplement: Supplementary file 1 [file lg5c00105_si_001.pdf]

Supporting Information: Topology-dependent polymer stretching and scission in solution at extreme shear rates

Bas G. P. van Ravensteijn,[†] Patrick T. Corona,[†] Anukta Datta,[†] Kexin Dai,[†] Raghida Bou Zerdan,[‡] Katie M. Weigandt,[¶] Ryan P. Murphy,[¶] Craig J. Hawker,[§] and Matthew E. Helgeson[*,†]

†*Department of Chemical Engineering, University of California Santa Barbara, Santa Barbara, CA 93106, USA*

‡*Mitsubishi Chemical Center for Advanced Materials, University of California Santa Barbara, Santa Barbara, CA 93106, USA*

¶*National Institute of Standards and Technology, Center for Neutron Research, Gaithersburg, Maryland, 20899, USA*

§*Materials Department, University of California, Santa Barbara, Santa Barbara, CA 93106, USA*

E-mail: helgeson@ucsb.edu

Contents

S1 Experimental Details **2**

S1.1 Materials 2

S1.2 Intrinsic Viscosity measurements 2

S1.3 Determination of relaxation times from capillary rheology 3
S1.4 Molecular weight and radius of gyration (Rg) determination 5
S1.5 Size exclusion chromatograms from *ex situ* shearing experiments 6
S1.6 Size exclusion chromatograms of polymers sheared in capillary set-up 8
S1.7 Static SANS with corresponding Guinier fits to determine $R_{g,eq}$ 9

S2 Scattering theoretical models 10
S2.1 Guinier-Maxwell model for scattering from Hookean dumbbell polymers in shear flow 10
S2.2 Guinier-Maxwell model for scattering from Hookean dumbbell polymers in capillary shear flow 14

S3 Estimating branching and functionality for a randomly branched polymer 15

S1. Experimental Details

S1.1 Materials

The poly(stearyl methacrylate-co methyl methacrylate) (p(SMA-co-MMA)) based polymer library ($LP_{170}, LP_{220}, BP_{130}, SP_{180}$) was synthesized and purified as described in our previous works[1]. A linear polystyrene standard ($Mw = 400kDa$) was purchased from PolyScience. Toluene (ACS grade), chloroform ($CHCl_3$, HPLC grade) and triethylamine (TEA, Reagent grade) were obtained from Fischer Scientific. Toluene-d_8 (D, 99.5%) for SANS measurements was purchased from Cambridge Isotopes. All chemicals were used as received.

S1.2 Intrinsic Viscosity measurements

The zero-shear dynamic viscosities ($\eta_{0,dyn}$) of dilute polymer solutions were measured using an Anton Paar Lovis 2000M rolling ball viscometer. The temperature controlled Lovis

module was equipped with a 1.5 mm steel ball in a glass capillary with an inner diameter of 1.59 mm. The rolling times of the ball were measured over a distance of 25 mm at an inclination angle of 30°. The measurements were performed at 25°C. The solution viscosities required to calculate the intrinsic viscosity ($[\eta]$) were averaged over at least 10 individual measurements.

Huggins and Kraemer plots were constructed by extrapolating the measured viscosities from the rolling ball viscometer at different concentrations in the dilute regime to $c = 0$ as shown in Figure S1.1.

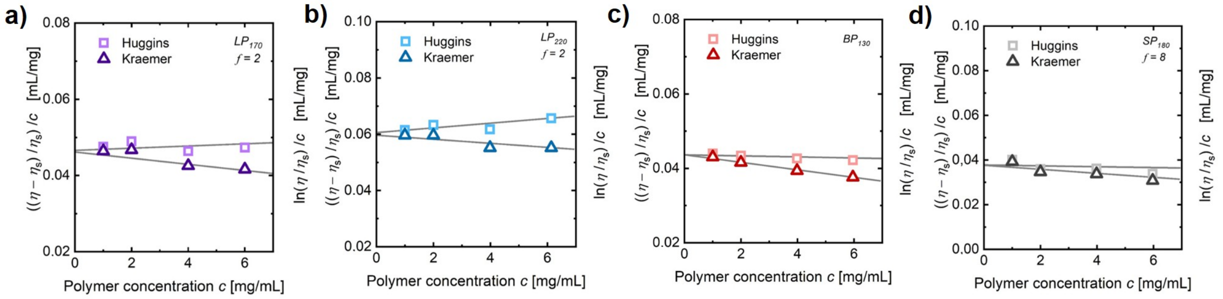

Figure S1.1: Huggins (squares) and Kraemer (triangles) extrapolations for a) LP_{170}, b) LP_{220}, c) BP_{130}, and d) SP_{180} p(SMA-*co*-MMA) polymer additives dissolved in toluene. η is the viscosity of the polymer solution, η_S is the viscosity of pure toluene, and c denotes the polymer concentration. The intrinsic viscosity ($[\eta]$) is found by extrapolation of the specific viscosity data to $c = 0$.

S1.3 Determination of relaxation times from capillary rheology

We used a similar set up of the capillary device as used for the CR-SANS measurements (see Main Text) to measure the steady-shear rheology of the complete polymer library (LP_{170}, LP_{220}, BP_{130}, SP_{180}; 3 wt% in toluene). The Cetoni Nemesys M syringe pumps used for these set of experiments have a maximum pressure drop of 200 bar for $3mL$ stainless steel syringes (lower than that used for the scattering measurements). All the measurements were done using a capillary of 100 μm inner diameter and a length of 38 cm and the lowest shear rates that we were able to probe was around $20,000$ s^{-1}. As shown in Figure S1.2, all polymers remain Newtonian until very high shear rates ($\sim 80,000$ s^{-1}) and only begin shear

thinning around 10^5 s^{-1}. Therefore, we are confident that the shear rate ranges accessible through these capillary devices are sufficient to capture the onset of shear thinning which was subsequently used to estimate the relaxation time of these polymers. The low-shear viscosities of the polymers were in good agreement with the zero-shear viscosities measured using a rolling ball viscometer at this concentration further showcasing the validity of this set-up for viscosity measurements. The power-law indices extracted from the Carreau-Yasuda fits are of -0.63, -0.55 and -0.56 for LP_{220}, LP_{170}, and SP_{180}. Error bars on the fit parameters (relaxation time, zero-shear viscosity and power law indices) were estimated from the standard errors of the fitted parameters obtained from the covariance matrix returned by the nonlinear least-squares fitting routine.

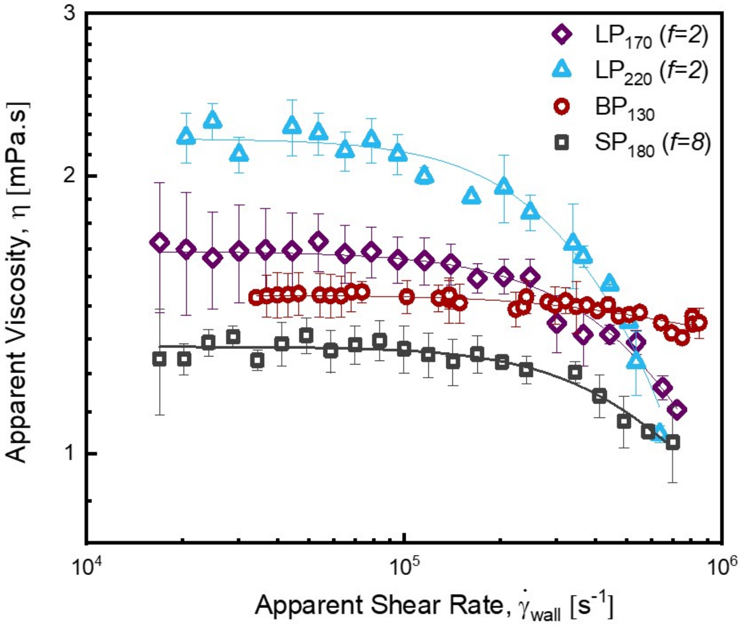

Figure S1.2: Steady-shear rheology of the p(SMA-*co*-MMA) polymer additives using the high-shear capillary rheometer along with their corresponding Carreau-Yasuda fits shown in solid lines.

We performed a Carreau-Yasuda model[2] fit to the viscosity (η) - shear rate ($\dot{\gamma}_{wall}$) data to estimate the relaxation times using the following formula-

$$\frac{\eta - \eta_\infty}{\eta_0 - \eta_\infty} = \left[1 + (\lambda\,\dot{\gamma}_{\rm wall})^2\right]^{\frac{n-1}{2}} \tag{S1.1}$$

where the fitting parameters are the infinite shear viscosity (η_∞), zero-shear viscosity (η_0), relaxation time of the polymer (λ), and the power law-index (n). The fitting was done using a nonlinear regression. The obtained results matched closely to theoretical relaxation times obtained for these polymers using the Zimm relaxation time in a good solvent[3],

$$\lambda_{Zimm} = \frac{1.25\eta[\eta_s]M_w}{RT} \tag{S1.2}$$

Table S1.1: Comparing the longest relaxation times (λ) obtained from experiments with theoretical values.

**Additives**	$\lambda_{Carreau-Fit}$-**Experimental** [μs]	λ_{Zimm}-**Theoretical** [μs]
LP_{170}	2.9 ± 0.063	2.6
LP_{220}	3.6 ± 0.039	3.6
BP_{130}	3.4 ± 0.045	3.2
SP_{180}	2.1 ± 0.081	2.0

S1.4 Molecular weight and radius of gyration (Rg) determination

The chromatography instrument (SEC-MALS) comprised a Waters Alliance HPLC 2695 separation module in combination with two 300×7.8 mm, 5 μm Agilent PolyPore SEC columns (flow rate = 1 mL/min) coupled to a Wyatt DAWN HELEOS-II light scattering detector (λ_0 = 663.1 nm) and a Wyatt Optilab rEX differential refractive index (dRI) detector. $CHCl_3$ with 0.25% TEA was used as the mobile phase. 100 μL of a polymer solution with known concentration ($3 - 5$ mg/mL) was injected for analysis. The resulting light scattering data were analyzed following a partial Zimm formalism developed for static light scattering (SLS) of dilute solutions of non-interacting polymers[4] -

$$\frac{K^* c}{R_\theta} = \frac{1}{M_w}\left(1 + \frac{16\pi^2}{3\lambda_0^2}\langle R_g^2\rangle \sin^2\left(\frac{\theta}{2}\right)\right) \tag{S1.3}$$

$$K^* = \frac{4\pi^2 n_0^2}{\lambda_0^4 N_A}\left(\frac{dn}{dc}\right)^2 \tag{S1.4}$$

In Eq. S1.3, c represents the polymer concentration, M_w is the absolute molecular weight, R_θ is the measured Rayleigh ratio, $\langle R_g^2 \rangle$ is the average radius of gyration squared, θ is the scattering/detector angle, K^* is an instrument-dependent constant depending on the wavelength of the laser (λ_0), n_0 is the refractive index of the mobile phase, $\frac{dn}{dc}$ is the refractive index increment and N_A represents Avogadro's number. $\frac{dn}{dc}$ values for the additives of interest were previously determined by injecting a concentration series of the individual polymers in the SEC-MALS instrument. Plotting the integrated dRI signal intensity against the injected polymer mass yielded linear curves with a slope equal to the $\frac{dn}{dc}$. This procedure yielded $\frac{dn}{dc}$ values of $0.0405, 0.0405$, and 0.0435 mg/mL for the linear, branched, and star-shaped polymers, respectively. Absolute molecular weights and R_g were evaluated at the retention time where the dRI signal reached its maximal value. According to Eq. S1.3, M_w, and R_g were obtained by plotting K^*c/R_θ versus $\sin^2(\frac{\theta}{2})$ (evaluated with at least 7 detector angles) and fitting the data with a linear relation ($R^2 \geq 0.93$ for all fits). The numerical values for the intercept with the y-axis, and the slope of the fitted curve yielded values for the absolute molecular weight and R_g, respectively (Main text, Table 1).

S1.5 Size exclusion chromatograms from *ex situ* shearing experiments

The size exclusion chromatograms (SECs) of the polymers sheared using high pressure homogenization were acquired on a Water Acquity Advanced Polymer Chromatography (APC) system equipped with an Acquity dRI detector and three 4.6 mm × 75 mm Water Acquity APC XT-extended temperature columns (pore diameters: 45 Å, 200 Å and 450 Å). The stationary phase consists of ethylene bridged hybrid particles (average diameter 1.7 μm for the 45 Å column, 2.5 μm for the 200 Å and 450 Å columns) prepared from high purity tetraethoxysilane (TEOS) and bis(triethoxysilyl) ethane (BTEE), resulting in mechanically resilient packings able to resist the 35×10^3 kPa operating pressure. The mobile phase consisted of $CHCl_3$ with 0.25% TEA flowing at 0.5 mL/min. The column temperature was fixed

at 35°C. Samples were filtered through 0.45 μm syringe filters before injection.

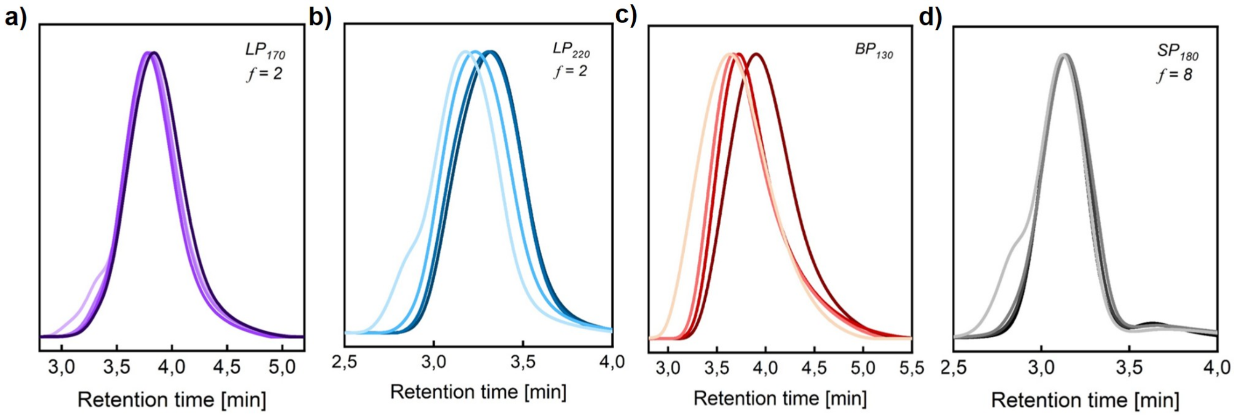

Figure S1.3: Molecular weight distributions as determined using size exclusion chromatography (SEC) as a function of the number of high pressure homogenization cycles for the a) $LP_{170}(f = 2)$, b) $LP_{220}(f = 2)$, c) BP_{130} and, d) $SP_{180}(f = 8)$. SEC traces were measured after 0, 10, 20, and 30 homogenization cycles

The resilience against mechanical degradation was probed by monitoring the evolution of the molecular weight distribution as function of shearing time using SEC for all p(SMA-co-MMA) additives as shown in Figure S1.3. Evidently, for LP_{220} the complete molecular weight distribution shifts to shorter retention times upon increasing the shearing time (Figure S1.3b), dark blue $\rightarrow$ light blue). An increase in retention time is a direct measure for a smaller coil volume and hence lower molecular weight of the polymeric species. We must note that the low retention time, i.e., high molecular weight shoulder in the molecular weight distributions originates from individual polymer chains that are coupled together during synthesis. Due to significantly higher molecular weight of these macromolecules compared to the main population, these species are most susceptible to chain breakage[5,6]. This high molecular weight shoulder therefore disappears during the initial shearing cycles. The same behavior is observed for the linear LP_{170} (Figure S1.3a) and the randomly branched BP_{130} (Figure S1.3c). In sharp contrast, the molecular weight distributions of the star-shaped additives are unaffected by applied flow conditions (Figure S1.3d).

S1.6 Size exclusion chromatograms of polymers sheared in capillary set-up

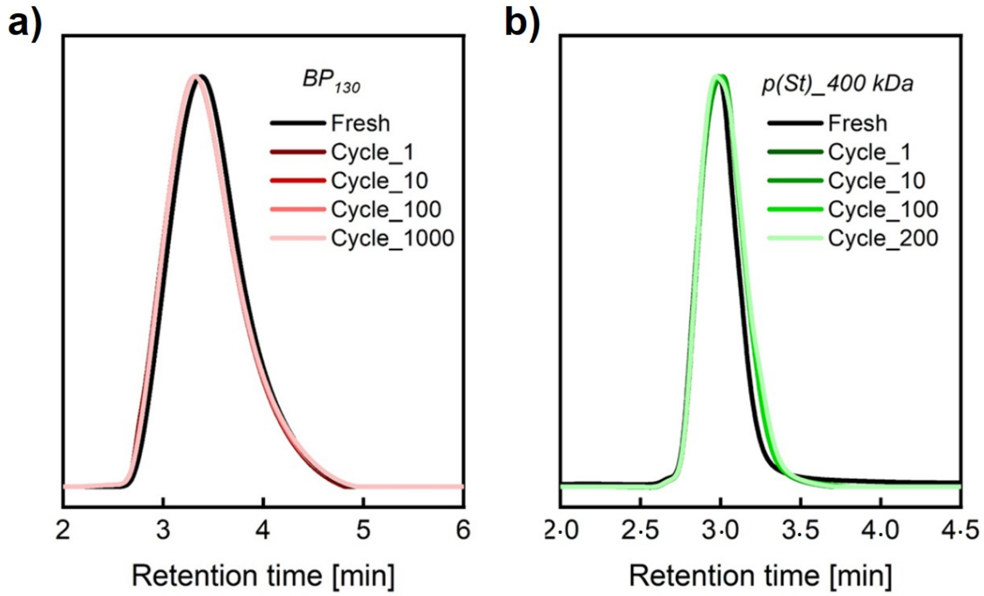

Figure S1.4: Molecular weight distributions as determined using size exclusion chromatography (SEC) as a function of the number of cycles through the CR-SANS set-up ($\dot{\gamma} = 0.5 \times 10^6$ s^{-1}) for the a) BP_{130} and b) a high molecular weight (400 kDa) polystyrene (p(St)) reference polymer.

We used the same SEC setup as described above to check if the polymer solutions in the capillary rheo-SANS measurements underwent chain scission (which would reduce the molecular weight). Figure S1.4 shows the molecular weight distribution for the branched polymer, BP_{130} and a high molecular weight polystyrene standard, $p(St)$, 400 kDa. The molecular weight of this linear polymer is significantly higher compared to the molecular weights of the p(SMA-co-MMA) additives of interest. Since no degradation was observed for this $p(St)$, we are confident chain scission can be neglected for the measurements on the polymer library probed throughout this study.

S1.7 Static SANS with corresponding Guinier fits to determine $R_{g,eq}$

Equilibrium radii of gyration ($R_{g,eq}$) for all polymers were extracted from 1D-SANS scattering experiments. The measurements were performed in the capillary set up in the absence of any flow. The same polymer solutions (3 wt% in d_8-toluene) were used as the ones used in the rheo-scattering measurements. The obtained scattering profiles are depicted in Figure S1.5 (experimental data are the open symbols). The low q-regime of the SANS profiles were fitted using the following Guinier expression:

$$I(q) = I(0)\exp(\frac{-q^2 R_{g,eq}^2}{3}) \tag{S1.5}$$

with the fitting parameters as, $I(0)$, the scattered intensity at $q = 0$, and $R_{g,eq}$, the radius of gyration of the polymer at equilibrium. The q-range used for this fitting was selected such that $qR_g \leq 1$ to ensure validity of this model. The resulting fits are plotted op top of the experimental data in Figure S1.5 in solid black lines, revealing satisfactory description of the low q data by the Guinier model. A summary of the fitted q-range and resulting $I(0)$ and $R_{g,eq}$ can be found in Table S1.2. Extracted $R_{g,eq}$'s from SANS were in close agreement with values measured using static light scattering, providing additional confidence in the validity of the SANS data and validity of the Guinier fitting procedure.

Table S1.2: Physical characteristics of employed poly(SMA-co-MMA)-based polymers

**Additives**	$R_{g,eq}$-**SEC** [nm]	$R_{g,eq}$-**SANS** [nm]	I_0-**SANS** [cm^{-1}]	**Guinier** q-**range** [Å^{-1}]
LP_{170}	11.6 ± 0.7	11.5 ± 4.9	0.073 ± 0.011	$0.01 - 0.025$
LP_{220}	17.7 ± 1.6	20.1 ± 4.9	0.096 ± 0.010	$0.0065 - 0.015$
BP_{130}	10.3 ± 1.6	11.4 ± 4.5	0.074 ± 0.011	$0.01 - 0.025$
SP_{180}	8.0 ± 0.6	8.5 ± 1.1	0.071 ± 0.010	$0.0125 - 0.025$

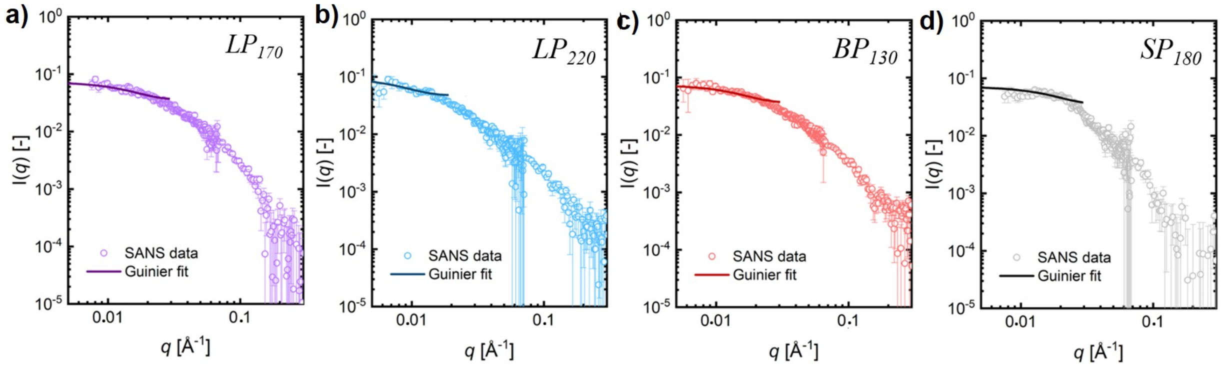

Figure S1.5: 1D small angle neutron scattering (SANS) profile for the a) LP_{170}, b) LP_{220}, c) BP_{130}, and d) SP_{180} p(SMA-*co*-MMA) additives dissolved in d_8-toluene. The measurements were performed in the capillary rheoSANS set-up in the absence of any shear ($\dot{\gamma} = 0$). The equilibrium radii of gyration ($R_{g,eq}$) were extracted from the data by fitting a Guinier expression to the low q-region (solid lines).

S2. Scattering theoretical models

S2.1 Guinier-Maxwell model for scattering from Hookean dumbbell polymers in shear flow

Modeling the scattering of isolated (dilute) polymers in the Guinier regime is accomplished by approximating the polymer coils as two infinitesimal beads of constant scattering length connected by an elastic spring with the same scattering length density as the solvent (Figure S2.1). This situation is the scattering equivalent of the elastic bead-spring dumbbell model used to model polymer stretch and implicitly assumes that the distribution of scattering density in the polymer is proportional to the end-to-end vector probability distribution function from an elastic dumbbell model. We consider this assumption to be approximately valid in the Guinier regime ($qR_g \leq 1$, where q is the scattering vector and R_g is the radius of gyration of the polymer chain), where scattering contributions due to more detailed chain architecture are negligible.

The diffraction from polymer chains is related to correlations between segments of the chain. In general, the coherent scattering is calculated as,

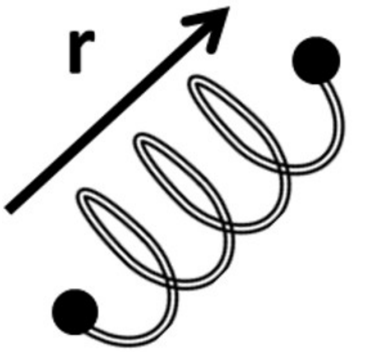

Figure S2.1: Pictorial representation of the combined micromechanical and scattering model for dilute polymer chains in flow. The black beads at the end of the coil are point sources of scattering density and hydrodynamic drag, which are enlarged in this picture to finite size, and are subject to thermal fluctuations. The coil connecting the two beads (outlined with a black line) has a scattering length density matching the surrounding solvent and a Hookean force vs. chain end-separation spring constant. The vector describing the separation of the chain ends (r) completely describes the chain's conformation in this model.

$$I(q) = \frac{I(0)}{N^2} \sum_{i=1}^{N} \sum_{j=1}^{N} \int e^{-i\mathbf{q}\cdot\mathbf{r}_{ij}} \, \psi_{ij}(\mathbf{r}_{ij}) \, d\mathbf{r}_{ij} \tag{S2.1}$$

where $I(0)$ is a constant prefactor related to the scattering density contrast in the system, N is the number of scattering points considered, r_{ij} is the vector describing the separation between the ith and jth scattering point, and $\psi_{ij}(r)$ is the conformation distribution function describing the probability of finding scatterers i and j a separation distance of r apart[7]. Note that this expression does not consider correlations between two polymer chains, for which one would also need to incorporate an inter-chain structure factor. One can factor out 'self' contributions to the scattering to yield,

$$I(q) = \frac{I(0)}{N^2} (N + \sum_{i=1}^{N} \sum_{j\neq i}^{N} \int e^{-i\mathbf{q}\cdot\mathbf{r}_{ij}} \, \psi_{ij}(\mathbf{r}_{ij}) \, d\mathbf{r}_{ij} \tag{S2.2}$$

In this work, we will consider the situation in Figure S2.1 where $N = 2$ and the conformation distribution between the two points is described by a simple Hookean dumbbell model. Simplifying the expression for $N = 2$ yields,

$$I(q) = \frac{I(0)}{2}\left(1 + \int e^{-i\mathbf{q}\cdot\mathbf{r}}\,\psi(\mathbf{r})\,d\mathbf{r}\right) \tag{S2.3}$$

where, r is the vector describing the separation between the beads, and $\psi(r) = \psi_{12}(r_{12}) = \psi_{21}(r_{21})$ is the conformation distribution function or the probability that chain ends are located at a separation distance r. Furthermore, $\psi(r)$ is normalized such that $\int \psi(r)dr = 1$. Again, this expression is only valid in the low-q region such that scattering due to intra-chain correlations do not vary with q. This will become an especially important consideration under non-equilibrium conditions when the polymer chain can be stretched causing the contribution of intra-chain correlations to extend to lower q as compared to equilibrium. The determination of conformation distribution functions for dilute polymer solutions has been explored extensively, and we will now consider the situation where analytical progress can be made for polymer solutions under flow.

For the situation of two thermal beads connected with a Hookean spring in a homogeneous flow field (see Figure S2.1), the conformation distribution function can be solved analytically for arbitrary flow histories as[2],

$$\psi(\mathbf{r},t) = \left(\frac{3}{\pi\langle R_g^2\rangle_{\text{eq}}\sqrt[3]{\det\boldsymbol{\alpha}(t)}}\right)^{3/2} \exp\left(-\frac{3}{\pi\langle R_g^2\rangle_{\text{eq}}}\,\boldsymbol{\alpha}^{-1}(t) : \mathbf{rr}\right) \tag{S2.4}$$

$$\boldsymbol{\alpha}(t) = \mathbf{I} - \frac{1}{\lambda}\int_{-\infty}^{t} e^{(t'-t)/\lambda}\left(\mathbf{I} - \mathbf{B}(t,t')\right)\,dt' \tag{S2.5}$$

where $\langle R_g^2\rangle_{eq}^{1/2}$ is the equilibrium center-of-mass radius of gyration, λ is the Hookean spring's relaxation time, $\mathbf{I}$ is the identity tensor, and $\mathbf{B}$ is the Finger strain tensor[8]. For

steady simple shear flow, the conformation distribution function reduces to,

$$\psi(x, y, z) = \left(\frac{3}{\pi \langle R_g^2 \rangle_{\text{eq}} \sqrt[3]{1 + \text{Wi}^2}} \right)^{3/2} \times \\ \exp \left(- \frac{3 \left(x^2 - 2\,\text{Wi}\, xy + (1 + 2\,\text{Wi}^2) y^2 + (1 + \text{Wi}^2) z^2 \right)}{\langle R_g^2 \rangle_{\text{eq}} (1 + \text{Wi}^2)} \right) \tag{S2.6}$$

where, x, y, and z are the coordinates of r in the flow, flow-gradient, and vorticity directions respectively and $Wi = \lambda\dot{\gamma}$ is the dimensionless Wiessenberg number defined in terms of the shear rate, $\dot{\gamma}$. Using this this conformation distribution function in the expression for the scattering intensity yields,

$$\bar{I}(q_u, q_{\nabla u}, q_\omega) = \frac{I(0)}{2} \left(1 + \exp \left[- \frac{\langle R_g^2 \rangle_{\text{eq}} \left(q_u^2 (1 + 2\,\text{Wi}^2) - 2\,\text{Wi}\, q_u q_{\nabla u} + q_{\nabla u}^2 + q_\omega^2 \right)}{3} \right] \right) \tag{S2.7}$$

where q_u, $q_{\nabla u}$, q_ω are the scattering vectors in the flow, flow-gradient, and vorticity directions. This is the **Guinier-Maxwell model for a Hookean dumbell under simple shear flow**. One can analyze the equilibrium scattering prediction when $Wi = 0$ to yield,

$$\bar{I}(q_u, q_{\nabla u}, q_\omega) = \frac{I(0)}{2} \left(1 + \exp \left[- \frac{\langle R_g^2 \rangle_{\text{eq}} \left(q_u^2 + q_{\nabla u}^2 + q_\omega^2 \right)}{3} \right] \right) \tag{S2.8}$$

that can be compared to the Guinier model,

$$\bar{I}(q) = I(0) \exp\left(\frac{- < R_g^2 >_{eq} q^2}{3} \right) + b \tag{S2.9}$$

where $q^2 = q_u^2 + q_{\nabla u}^2 + q_\omega^2$, $\langle R_g^2 \rangle$ is the center of mass radius of gyration, and b is the incoherent background scattering. By inspection, one can see that the prediction under simple shear flow reduces to the Guinier model when $b = I(0)/2$ and $Wi = 0$ or more generally when $\alpha = I$ (i.e. no flow). This matches our expectation about the validity of the

model being restricted to the low-q region.

S2.2 Guinier-Maxwell model for scattering from Hookean dumbbell polymers in capillary shear flow

In the capillary-rheo SANS experiment, the measured scattering represents a macroscopic average of the scattering from all parts of the capillary illuminated by the neutron beam. This means that the measured scattering probes polymer conformations under shear rates ranging from a maximum at the wall to zero at the capillary center. Assuming the velocity field in the capillary is well developed (steady state flow), laminar pipe flow of a Newtonian fluid, the shear rate variation through the capillary is linear varying from 0 to $\dot{\gamma}_{max}$ from the centerline to wall. Additionally, due to the cylindrical symmetry in the capillary, the probing radiation is incident with equal likelihood in the flow-gradient and vorticity planes, while the flow direction is always in the $\pm q_y$ direction on the detector. We can utilize the cylindrical symmetry to define the laboratory coordinate system as $q_u = q_y$, $q_{\nabla u} = q_x cos(\theta) + q_z sin(\theta)$, and $q_\omega = q_x sin(\theta) + q_z cos(\theta)$ where q_x and q_y are the scattering vector positions measured on the 2D detector, q_z is the direction of the incident radiation (which is set to zero because the sample-to-detector distance is much greater than the size of the detector), and θ is the angle between the incident radiation and the vorticity direction. Combining these considerations produces the expression for the predicted average coherent scattering in the capillary rheo-SANS device from the Guinier-Maxwell model -

$$\bar{I}(q_x, q_y) = c\Bigg(1 + \frac{2}{\pi\,\mathrm{Wi}_{\mathrm{max}}^2}\int_0^{2\pi}\int_0^{\mathrm{Wi}_{\mathrm{max}}} \mathrm{Wi}\cdot\exp\Bigg[-\frac{\langle R_g^2\rangle_{\mathrm{eq}}}{3}\Big((q_x\cos\theta)^2 + (q_x\sin\theta)^2 + q_y^2(1+2\,\mathrm{Wi}^2) - 2\,\mathrm{Wi}\,q_y q_x\cos\theta\Big)\Bigg]\,d\mathrm{Wi}\,d\theta\Bigg) \tag{S2.10}$$

where Wi_{max} is the Weissenberg number at the capillary wall, Wi_{wall} ($Wi_{wall} = \dot{\gamma}_{wall}.\lambda$ (and, maximum shear rate in the capillary is the shear rate at the capillary wall, $\dot{\gamma}_{wall}$). Note that the integrating factor of Wi is included because Wi is a function of the distance from the centerline, r through the shear rate. The integration over θ can be carried out analytically yielding,

$$\bar{I}(q_x, q_y) = I(0)\left(1 + \frac{4}{\mathrm{Wi}_{\mathrm{wall}}^2}\int_0^{\mathrm{Wi}_{\mathrm{wall}}} \mathrm{Wi}\cdot\exp\left(-\frac{\langle R_g^2\rangle_{\mathrm{eq}}(q_x^2 + q_y^2(1+2\,\mathrm{Wi}^2))}{3}\right) \times I_0\left(\frac{2}{3}\mathrm{Wi}\,\langle R_g^2\rangle_{\mathrm{eq}}\,q_x q_y\right)\,d\mathrm{Wi}\right) \tag{S2.11}$$

where I_0 is the modified Bessel function of the first kind. This expression is solved numerically to predict the scattering at corresponding q_x, q_y coordinates on the detector for specified $I(0)$, Wi_{wall} and $\langle R_g^2\rangle_{eq}$ for comparison to CR-SANS experiments.

S3. Estimating branching and functionality for a randomly branched polymer

We seek to determine the degree of branching of the randomly branched polymer used in this work, and therefore $\langle f_{eff}\rangle$. As we will show, the problem reduces to determining the expected average span molecular weight of a randomly branched polymer comprised of N_{tot} monomers. The polymer is formed by uncontrolled radical polymerization, in which monomers predominately extend the backbone linearly, but will infrequently produce three-fold branch points with a probability p per monomer due primarily to chain transfer reactions. This process produces chains with a random number of branch points, n_{br}, resulting in branches that contain a random number N_{br} of monomers, leaving N_{span} monomers in the longest linear chain between its ends. The number of branch points along a chain of length N_{span} is therefore a Poisson variable, with probability density given by a Poisson distribution,

$$n_{\mathrm{br}}(N_{\mathrm{span}}) = \frac{p^{N_{\mathrm{span}}}\, e^{-N_{\mathrm{span}}}}{(N_{\mathrm{span}}-1)!} \tag{S3.1}$$

The average number of branches per chain is then given by the expectation value of this distribution,

$$\langle n_{\mathrm{br}}\rangle = \int_0^{N_{\mathrm{span}}} n_{\mathrm{br}}(N)\, dN = pN_{\mathrm{span}} \tag{S3.2}$$

The span molecular weight is constrained by a total monomer balance,

$$N_{tot} = \langle N_{span}\rangle + \langle n_b\rangle\langle N_{br}\rangle \tag{S3.3}$$

where $\langle N_{br}\rangle$ is the average number of monomers in a branch chain. To predict $\langle N_{br}\rangle$, we consider that the growth of the branching chains is itself also a Poisson process where the probability that a branched chain grows to become N_{br} monomers is given by the joint probability that a reacting monomer is added to a particular chain and that the chain does not branch after N_{br} monomers. Given this, the expectation value $\langle N_{br}\rangle$ is then given by,

$$\langle N_{br}\rangle = \frac{(1-p)N_{tot}}{1+p\langle N_{span}\rangle} \tag{S3.4}$$

Using the results above, we obtain the following estimate for the average span molecular weight,

$$\langle N_{\mathrm{span}}\rangle = \frac{2(1-p)}{1-p^2N_{\mathrm{tot}} + \sqrt{N_{\mathrm{tot}}^2p^4 - 2N_{\mathrm{tot}}(p-2)p+1}} \tag{S3.5}$$

For a star polymer with number of arms f, the functionality requires that $f = \frac{2N_{tot}}{\langle N_{span}\rangle}$. Thus, for a randomly branched polymer, the equivalent functionality compared to a star of the same span molecular weight is $\langle f_{eff}\rangle = \frac{2N_{tot}}{\langle N_{span}\rangle}$. Thus,

$$\langle f_{\mathrm{eff}}\rangle = \frac{1-p^2N_{\mathrm{tot}} + \sqrt{N_{\mathrm{tot}}^2p^4 - 2N_{\mathrm{tot}}(p-2)p+1}}{1-p} \tag{S3.6}$$

For the randomly branched polymer considered in this work (which has an average monomer molar mass of 219.3 g/mol), we estimate $N_{tot} \approx 1000$. For uncontrolled free radical homopolymerization of a mono-functional monomer, branching is assumed to occur primarily through chain transfer reactions. In this case, multiple studies have characterized the so-called "chain transfer constant", C_s, as a measure of the probability of chain transfer. In the nomenclature used here, $p \approx C_s^{-1}$. Basu *et al.*[9] report $C_s \sim 10^{-4}$–10^{-3} for AIBN-initiated radical polymerization of MMA under the relevant synthesis conditions. From this and the figure above, we therefore estimate $\langle f_{eff} \rangle = 2 - 3$. Thus, to facilitate further calculations, we choose $\langle f_{eff} \rangle \approx 2.5$.

The relatively small value of f that we find can be rationalized by the polymerization chemistry. Since the monomers MMA and SMA are mono-functional, they would normally yield linear chains, and branching can only arise through chain transfer to growing polymer chains. However, 1-dodecanethiol was added as a chain transfer agent, and chain transfer to this small molecule is far more likely than chain transfer to a polymer backbone. This process regulates the molecular weight while simultaneously suppressing the probability of intermolecular transfer events that generate branch points. Thus, in the absence of multi-functional monomers and with the presence of an efficient chain transfer agent, the random branching density is expected to be very low, consistent with the small f value extracted from our analysis.

References

(1) Ravensteijn, B. G. P. v.; Zerdan, R. B.; Seo, D.; Cadirov, N.; Watanabe, T.; Gerbec, J. A.; Hawker, C. J.; Israelachvili, J. N.; Helgeson, M. E. Triple Function Lubricant Additives Based on Organic–Inorganic Hybrid Star Polymers: Friction Reduction, Wear Protection, and Viscosity Modification. *ACS Applied Materials & Interfaces* **2018**, *11*, 1363–1375.

(2) Bird, R. B.; Armstrong, R. C.; Hassager, O. *Dynamics of polymeric liquids. Vol. 1. : Fluid mechanics*, 2nd ed.; John Wiley and Sons Inc.,New York, NY, 1987; Vol. 1.

(3) Zimm, B. H. Dynamics of Polymer Molecules in Dilute Solution: Viscoelasticity, Flow Birefringence and Dielectric Loss. *The Journal of Chemical Physics* **1956**, *24*, 269–278.

(4) Zimm, B. H. The Scattering of Light and the Radial Distribution Function of High Polymer Solutions. *The Journal of Chemical Physics* **1948**, *16*, 1093–1099.

(5) Odell, J. A.; Keller, A.; Rabin, Y. Flow-induced scission of isolated macromolecules. *The Journal of Chemical Physics* **1988**, *88*, 4022–4028.

(6) Xue, L.; Agarwal, U. S.; Lemstra, P. J. Shear Degradation Resistance of Star Polymers during Elongational Flow. **2005**,

(7) Hammouda, B. SANS from homogeneous polymer mixtures: A unified overview. *Advances in Polym. Sci.* **1993**, *106*, 87–133.

(8) Bird, R.; Curtiss, C.; Armstrong, R.; Hassager, O. *Dynamics of Polymeric Liquids, Volume 2: Kinetic Theory, 2nd Edition*, 2nd ed.; Wiley, 1987; Vol. 2.

(9) Basu, S.; Nath Sen, J.; R. Palit, S. Degree of polymerization and chain transfer in methyl methacrylate. *Proceedings of the Royal Society of London. Series A. Mathematical and Physical Sciences* **1950**, *202*, 485–498.
